# Complete genome of *Enterobacter sichuanensis* strain SGAir0282 isolated from air in Singapore

**DOI:** 10.1186/s13099-020-00350-z

**Published:** 2020-02-27

**Authors:** Akira Uchida, Hie Lim Kim, Rikky W. Purbojati, Vineeth Kodengil Vettath, Anjali B. Gupta, James N. I. Houghton, Caroline Chénard, Anthony Wong, Megan E. Clare, Kavita K. Kushwaha, Deepa Panicker, Alexander Putra, Cassie E. Heinle, Balakrishnan N. V. Premkrishnan, Ana Carolina M. Junqueira, Daniela I. Drautz-Moses, Stephan C. Schuster

**Affiliations:** 1 Singapore Centre for Environmental Life Sciences Engineering, Nanyang Technological University, Singapore, Singapore; 2 Asian School of the Environment, Nanyang Technological University, Singapore, Singapore; 3 Departamento de Genética, Instituto de Biologia, Universidade Federal Do Rio de Janeiro, Rio de Janeiro, Brazil; 4 School of Biological Sciences, Nanyang Technological University, Singapore, Singapore

**Keywords:** Airborne bacteria, *Enterobacter cloacae* complex, Whole genome sequencing, Multidrug resistant

## Abstract

**Background:**

*Enterobacter cloacae* complex (ECC) bacteria, such as *E. cloacae*, *E. sichuanensis*, *E. kobei*, and *E. roggenkampii*, have been emerging as nosocomial pathogens. Many strains isolated from medical clinics were found to be resistant to antibiotics, and in the worst cases, acquired multidrug resistance. We present the whole genome sequence of SGAir0282, isolated from the outdoor air in Singapore, and its relevance to other ECC bacteria by in silico genomic analysis.

**Results:**

Complete genome assembly of *E. sichuanensis* strain SGAir0282 was generated using PacBio RSII and Illumina MiSeq platforms, and the datasets were used for de novo assembly using Hierarchical Genome Assembly Process (HGAP) and error corrected with Pilon. The genome assembly consisted of a single contig of 4.71 Mb and with a G+C content of 55.5%. No plasmid was detected in the assembly. The genome contained 4371 coding genes, 83 tRNA and 25 rRNA genes, as predicted by NCBI’s Prokaryotic Genome Annotation Pipeline (PGAP). Among the genes, the antibiotic resistance related genes were included: Streptothricin acetdyltransferase (SatA), fosfomycin resistance protein (FosA) and metal-dependent hydrolases of the beta-lactamase superfamily I (BLI).

**Conclusion:**

Based on whole genome alignment and phylogenetic analysis, the strain SGAir0282 was identified to be *Enterobacter sichuanensis*. The strain possesses gene clusters for virulence, disease and defence, that can also be found in other multidrug resistant ECC type strains.

## Background

Species belonging to *Enterobacter cloacae* complex (ECC) are commonly found in environment [1, 2], and are widely known to be opportunistic pathogens. In the past decades, ECC such as *E. hormaechei*, *E. sichuanensis*, *E. asburiae*, *E. kobei*, and *E. roggenkampii*, have been a global health concern because of wide-spread antibiotic resistances and new acquisition of multidrug resistances [3–6]. They are intrinsically resistant to beta-lactam antibiotics, which are the most commonly used antibiotics such as ampicillin, amoxicillin and first generation cephalosporins [7].

Carbapenems are reserved to treat infections caused by multidrug resistant bacteria including ECC. An increasing number of patients that have been infected with these nosocomial pathogens can no longer be successfully treated due to the ECC’s acquired resistances to carbapenems and other classes of antibiotics after long term exposure to the drugs. It has been reported that the gut microbiome community of healthy volunteers administered with cefprozil, one of beta-lactam antibiotics, drifts towards a higher abundance of antibiotic resistant bacteria including ECC [8]. The affected gut microbiota can end up in the sewage of hospitals, and turn the wastewater facilities into a reservoir for antibiotic resistant bacteria [9, 10]. As such, there is an increasing risk of spreading multidrug resistant strains and associated genes by releasing them into the environment through wastewater and other means [10].

In this study, we report the complete genome sequence of the strain SGAir0282, a bacterium that was isolated from outdoor air at a university sports field. Using the whole-genome dataset, we identified its taxon as a recently defined species, *E. sichuanensis*, which is a member of the ECC. To our knowledge, this is the first report that ECC was isolated from air. Considering the importance of the studies on ECC that were mostly isolated from medical clinics/hospitals and reported as multidrug resistant species, the high-quality genome sequence of strain SGAir0282 from our study will make a significant contribution to this field.

## Methods

### Isolation and culture conditions

We generated a large-scale isolate collection of airborne microorganisms using cultivation methods to produce whole-genome sequence data of those isolates. The genome data is to improve the taxonomy identification of metagenomic data of air microbiome [11]. To have the vast diversity of the airborne microbiota, we used various media for agar plates as many as possible, and sampled in different time and locations. Air samples were collected with an Single-staged Andersen-type air sampler (SKC, USA). A total volume of 23.4 L of air was drawn against the agar plates over 2 minutes.

Strain SGAir0282 was isolated from outdoor air (global position system coordinates 1.349° N, 103.689° E) by impacting air onto a Potato Dextrose Agar (PDA, Sigma-Aldrich, USA), and the plate was aerobically incubated until colony formation at 30 °C. A single colony was picked and further streaked on Tryptic Soy Agar (Becton Dickinson, USA) to obtain a pure clonal colonies. One of these successfully sub-cultured colonies was the strain SGAir0282, which was then cultured in Lysogeny Broth media (Merck, USA) overnight at 30 °C followed by DNA extraction.

### DNA extraction and sequencing

DNA was extracted from an overnight culture of the isolate using the Wizard Genomic DNA Purification Kit (Promega, USA), according to the manufacturer’s recommended protocol. Sequencing was performed on the Pacific Biosciences (PacBio) RSII sequencer, using a library that was prepared with SMRTbell Template Prep Kit 1.0 (Pacific Biosciences, USA). The library used for sequencing on the Illumina MiSeq was prepared with the TruSeq Nano DNA Library Preparation Kit (Illumina, USA).

### Genome assembly and annotation

De novo assembly was performed with the Hierarchical Genome Assembly Process (HGAP) version 3 [12], which is part of PacBio SMRT Analysis 2.3.0 package and subsequently polished with Quiver [12]. Error correction was performed with Pilon version 1.16 using 300-bp MiSeq paired-end reads and following parameters (–tracks –changes –vcf –fix all –mindepth 0.1 –mingap 10 –minmq 30 –minqual 20 –K 47) [13]. Finally, the genome sequence was circularized using Circulator version 1.1.4 [14]. Gene prediction was carried out with NCBI’s Prokaryotic Genome Annotation Pipeline (PGAP) version 4.2 [15]. Rapid Annotations using Subsystems Technology tool kit (RASTtk) was used for additional feature annotation of the assembled contig [16]. Default parameters were used for all steps unless otherwise stated above.

### Genome sequence analysis for taxonomy identification

Average Nucleotide Identity (ANI) was calculated to identify the strain SGAir0282 to species level with a custom PERL script against all 10,744 bacterial genomes in the NCBI Reference Sequence Database (RefSeq; downloaded in April 2019) [17].

We also performed NCBI’s BLASTn using query sequence of 16S rRNA of the SGAir0282 against nucleotide (nt) database with default settings. Based on the BLASTn result, we retrieved whole-genome sequence of every strain which met following two criteria. Firstly, the 16S rRNA gene sequence had high similarity to the one from strain SGAir0282 (higher than 99.5% and 100% query coverage). Secondly, the level of genome assembly was ‘complete genome’ or ‘chromosome’, according to the NCBI nucleotide database. As exceptional, we included genome sequence of *E. sichuanensis* WCHECL1597 (BLASTn: 99.4% similarity and 99% query coverage) to analysis, even the genome assembly quality was not high enough, because *E. sichuanensis* reference genome showed the highest value of ANI to strain SGAir0282. In total, 27 whole-genome datasets, including SGAir0282, were used for further genome analyses below.

Core genome alignment was performed on Parsnp [18] with SGAir0282 as a reference. Gingr [18] was used to visualise aligned genomes and to export the Newick format of a phylogenetic tree reconstructed by Parsnp. The tree was plotted by an online tool, phylogenetic tree viewer (http://etetoolkit.org/treeview/) [19]. MASH was used to calculate ANI between SGAir0282 and other strains [20]. The genome datasets were individually processed by sketching prior to estimating the distance from SGAir0282. We applied kSNP3 [21] to identify single nucleotide polymorphisms (SNPs) and to obtain a distance matrix. The k-mer size was optimized as 19 nucleotides, calculated by a kSNP3-packaged program, kChooser. For annotation of SNPs, SGAir0282 was used as a reference sequence. Variant call format (VCF) generated by kSNP3 was attached as an Additional file 1. Parameters which were not mentioned above were kept at the default value.

Sequence Types (STs) were assigned to the ECC complex strains using multilocus sequence type (MLST) databases (https://pubmlst.org/ecloacae/) [22].

### Quality assurance

The assembly produced only a single contig and no trace of contamination of other microorganisms was found. Whole genome sequencing was carried out on two sequencing platforms (PacBio RSII and Illumina MiSeq) and led to assembly completion with a circularized chromosome.

## Results

HGAP de novo assembly was performed with 53,503 PacBio subreads. The assembly was polished with Quiver and corrected by aligning 858,191 paired-end short reads using Pilon version 1.16. The resulting assembly consisted of a single contig with a total size of 4,711,389 bp, and a mean genome coverage of 84.8- and 110-fold by PacBio and Illumina data, respectively. Chromosomal G+C content was 55.5%. Species identification with ANI analysis resulted in 98.7% similarity to *E. sichuanensis*. BLASTn alignment analysis using 16S rRNA gene of SGAir0282 showed high similarity to *E. cloacae* strain A1137 with 99.8% identity [23] and *E. sichuanensis* WCHECL1597 [3] with 99.4% identity (Additional file 2: Table S1).

We selected the 26 genomes, which are most closely related strains to SGAir0282, based on 16S rRNA genes (Additional file 2: Table S1) and quality of their genome assembly, as described in the method section. The resulting 27 genomes, including SGAir0282, were aligned, and the genetic distance between them were estimated using three different methods.

**Fig. 1:**
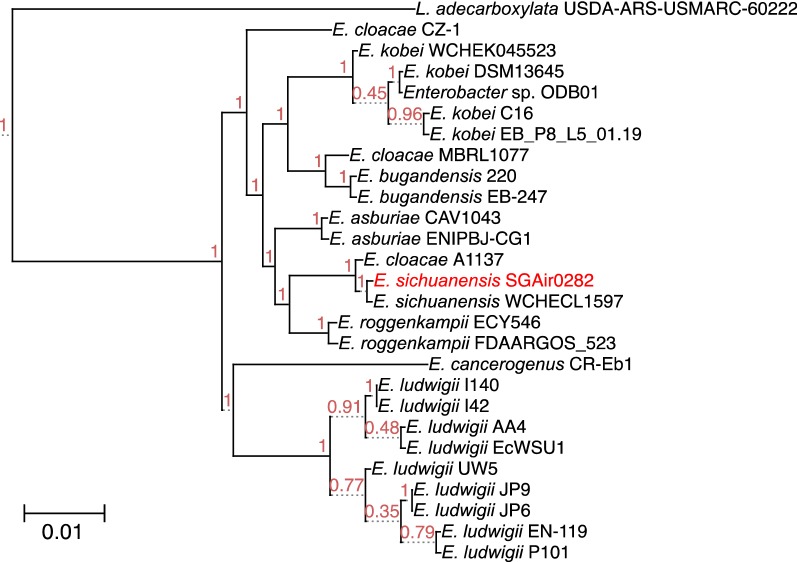
Phylogenetic tree of *E. sichuanensis* strain SGAir0282 and closely related strains by 16S rRNA gene sequence. Phylogenetic tree reconstructed by Parsnp was visualized and exported a Newick file with Gingr. Phylogenetic tree viewer (http://etetoolkit.org/treeview/) was used to plot phylogenetic tree. Number at joint indicated bootstrap values

The result produced by Parsnp [18] was presented as a phylogenetic tree (Fig. 1). In the tree, SGAir0282 was closest to *E. sichuanensis* WCHECL1597, and second closest to *E. cloacae* A1137 (Fig. 1). The estimated ANI [20] between SGAir0282 and each of the other 26 strains was consistent with the tree. The strain WCHECL1597 showed the smallest distance (0.014) to the stain SGAir0282, and the second shortest strain was the strain A1137 (0.019) (Table 1, Additional file 3: Table S2). Lastly, the distance matrix calculated by kSNP3 [21] also clearly supported the close relationships with the strains WCHECL1597 and A1137 (Additional file 4: Table S3). The robust and high similarity with *E. sichuanensis* WCHECL1597 suggests that SGAir0282 belongs to the species, *E. sichuanensis*.

**Table 1:** Genetic distance by MASH and allele and sequence type number by MLST Sequence type of the house-keeping genes were defined through PubMLST.org using database for *E. cloacae*. One strain from each species were listed

	Distance from ref.^d^	*p-*value	*dnaA*	*fusA*	*gyrB*	*leuS*	*pyrG*	*rplB*	*rpoB*	ST
SGAir0282	–	–	153	98	170	440^a^	344^a^	68	217	1330^a^
*E. sichuanensis* WCHECL1597	0.014	0	153	98	n.d.^c^	159	n.d.^c^	68	n.d.^c^	472^b^
*E. cloacae* A1137	0.019	0	241	98	170	287	237	68	109	738
*E. asburiae* ENIPBJ-CG1	0.083	0	347	15	39	134	47	10	113	n.d.^c^
*E. ludwigii* I140	0.103	0	13	2	n.d.^c^	286	51	2	13	n.d.^c^
*E. kobei* WCHEK045523	0.096	0	43	3	66	314	3	16	168	910
*E. ludwigii* AA4	0.104	0	15	158	365	416	55	105	222	1281
*E. bugandensis* 220	0.091	0	322	110	200	30	120	76	28	1140
*E. roggenkampii* ECY546	0.077	0	120	25	127	90	49	12	20	466

^a^New allele number and sequence type were given by curation

^b^The profile was suggested for nearest match with combination of allele by PubMLST.org

^c^n.d.: not defined. Database did not have exact allele and ST matches

^d^SGAir0282 was used as reference and each strain was used as a query sequence on MASH software

In the previous report [23], the strain A1137 belonged to ECC, based on a phylogenetic tree reconstructed for a single gene. Our analyses suggested the strain A1137 to be *E. sichuanensis*, as well as SGAir0282.

MLST was assigned for the strain SGAir0282 (Table 1). SGAir0282 had new alleles of *leuS* and *pyrG* genes, while for the other three genes, *dnaA*, *fusA* and *rplB*, it shared the same allele with the strain WCHECL1597. Therefore, SGAir0282 created a new ST (1330) in the database.

The SGAir0282 genome includes a total of 4611 genes, of which 4371 were coding genes. Ribosomal RNAs were reported as 9 copies of 5S and 8 copies each of 16S and 23S. Transfer RNAs were annotated with 83 genes. No evidence of plasmid DNA sequence was found.

**Fig. 2:**
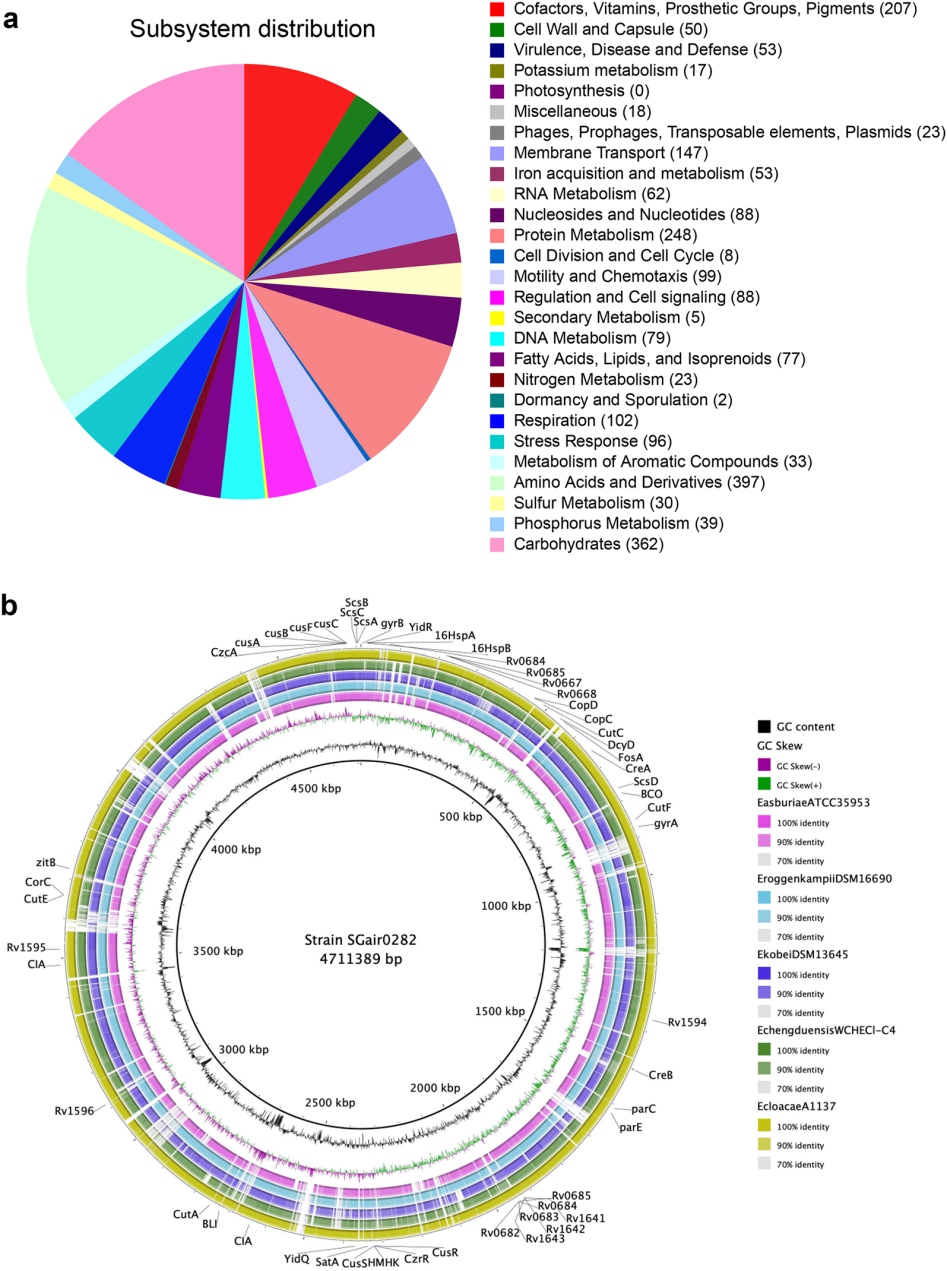
Gene annotation for *E. sichuanensis* strain SGAir0282 by RASTtk analysis. **a** Subsystem distribution of *E. sichuanensis* SGAir0282 based on RAST annotation. Number of annotated genes are indicated at each subsystem name. **b** Circular genomic map of selected genome of ECC type strain and strain A1137. BRIG was used to draw a map of genes predicted in virulence, disease and defence subsystem [25]. Fifty-three genes were annotated under the subsystem of the strain SGAir0282 as a reference. *E. sichuanensis* WCHECL1597 was not able to included due to genome assembly at contig level

As shown in Fig. 2a, RASTtk gene classification was estimated that SGAir0282 contained 53 genes, which were related to virulence, disease and defence. The 53 genes were shared across pathogenic ECC type strains, and their loci are indicated in Fig. 2b. The gene products conferring antibiotic resistance in the SGAir0282 genome were predicted: streptothricin acetyltransferase (SatA), fosfomycin resistance protein (FosA) and metal-dependent hydrolases of the beta-lactamase superfamily I (BLI). Strain SGAir0282 is potentially pathogenic due to the presence of virulence genes that are commonly found in pathogenic ECC type strains.

## Discussion

*Enterobacter sichuanensis* strain SGAir0282 was isolated from air at a side of sports and recreation field where high human activity was observed. The fact that this genus commonly found in soil [1, 2, 24], may suggest that this isolate was stirred up from ground and dispersed into the air prior to being captured onto the agar plate. Hence, our study indicates that environmental bacteria can be aerosolized, underlying that environmental microorganisms can be transported through wind in a form of aerosols.

The strains WCHECL1597 [3] and A1137 [23] were isolated from a chronic renal insufficiency patient’s urine (2013) and pneumonia patient’s blood (2016), respectively, in the hospitals in China. Those two patients were individually administered with antibiotics for the infections by other bacteria, and the two strains managed to acquire multidrug resistance through sets of antibiotic treatments. Multidrug resistant bacteria that have been released through facilities such as wastewater handling facilities of hospitals [10], are potentially aerosolized and transported through air or humans that host the strain of non-virulent bacteria as part of their microbiome.

The high similarity of SGAir0282 to these two strains clearly suggests that the strain SGAir0282 is a potential multidrug-resistant pathogen. This hypothesis is also supported by fact that SGAir0282 possesses metal-dependent hydrolases of the beta-lactamase superfamily I gene which confers resistance to beta-lactam antibiotics. Although the carbapenemase gene was not detected in the SGAir0282 genome, the strain could acquire a gene encoding carbapenemase by mutation and/or recombination, in the presence of carbapenem.

Our analysis emphasized that the importance of using whole genome data for the taxonomy identification. The most commonly used method for the taxonomy identification with a single gene showed low resolution due to the limited amount of data (Additional file 2: Table S1). With the whole genome sequences, our analyses revealed that the strain SGAir0282, *E. cloacae* A1137 and *E. sichuanensis* WCHECL1597 are closely related with a sequence similarity that is high enough to support the claim that these are the same species (Fig. 1, Additional file 3: Table S2 and Additional file 4: Table S3).

The genome assembly for *E. sichuanensis* WCHECL1597 is highly fragmented and consists of 204 contigs as of December 2019. On the other hand, the genome assembly of *E. sichuanensis* SGAir0282 consists of a single, circular chromosome with high coverage sequence quality, and we therefore suggest that this genome should be used as the reference sequence for *E. sichuanensis* species.

## Supplementary information

**Additional file 1.** The variant call was performed by kSNP3. Dataset used here was defined in the method section. Strain SGAir0282 was used as reference genome.

**Additional file 2: Table S1.** Similarity search result using 16S rRNA gene of strain SGAir0282. NCBI’s BLASTn search was used for submitting query sequence, 16S rRNA gene. The alignment search was performed against nucleotide collection. Other parameters were kept at default.

**Additional file 3: Table S2.** Pairwise distance from strain SGAir0282 estimated by MASH. Pairwise distance of Reference sequence (Ref seq) against Query sequence (Query seq) was estimated. The same dataset was used as Fig. 1. Default setting was used for this analysis.

**Additional file 4: Table S3.** Pairwise distance matrix showing distance between genomes on the Neighbour-joining tree. k-mer based kSNP3 analysis was performed on our dataset. k-mer was set at 19 nucleotides. Genome sequence of SGAir0282 was used as reference.

## Data Availability

The complete genome sequence of *Enterobacter sichuanensis* SGAir0282 has been deposited in DDBJ/EMBL/GenBank under the accession numbers CP027986.
