# Supplementary material for: Complete genome of Enterobacter sichuanensis strain SGAir0282 isolated from air in Singapore
Source: Gut Pathog. 2020 Feb 27;12:12. doi: 10.1186/s13099-020-00350-z (PMC7045367; doi:10.1186/s13099-020-00350-z)
Supplement: Supplementary file 3 — Additional file 3: Table S2. Pairwise distance from strain SGAir0282 estimated by MASH. Pairwise distance of Reference sequence (Ref seq) against Query sequence (Query seq) was estimated. The same dataset was used as Fig. 1. Default setting was used for this analysis. [file 13099_2020_350_MOESM3_ESM.docx]

| Ref seq | Query seq | Mash-Distance | P-value | matching-hashes |
| --- | --- | --- | --- | --- |
| EcloacaeA1137.fasta | SGAir0282.fasta | 0.01906 | 0 | 504/1000 |
| EsichuanensisWCHECL1597.fasta | SGAir0282.fasta | 0.01440 | 0 | 586/1000 |
| EasburiaeCAV1043.fasta | SGAir0282.fasta | 0.08674 | 0 | 88/1000 |
| EasburiaeENIPBJ-CG1.fasta | SGAir0282.fasta | 0.08295 | 0 | 96/1000 |
| Ebugandensis220.fasta | SGAir0282.fasta | 0.09093 | 0 | 80/1000 |
| EbugandensisEB-247.fasta | SGAir0282.fasta | 0.09320 | 0 | 76/1000 |
| EcancerogenusCR-Eb1.fasta | SGAir0282.fasta | 0.12827 | 2.34E-155 | 35/1000 |
| EcloacaeCZ-1.fasta | SGAir0282.fasta | 0.09685 | 0 | 70/1000 |
| EcloacaeMBRL1077.fasta | SGAir0282.fasta | 0.09093 | 0 | 80/1000 |
| EkobeiC16.fasta | SGAir0282.fasta | 0.09438 | 0 | 74/1000 |
| EkobeiDSM13645.fasta | SGAir0282.fasta | 0.09320 | 0 | 76/1000 |
| EkobeiEB_P8_L5_01.19.fasta | SGAir0282.fasta | 0.09622 | 0 | 71/1000 |
| EkobeiWCHEK045523.fasta | SGAir0282.fasta | 0.09559 | 0 | 72/1000 |
| EludwigiiAA4.fasta | SGAir0282.fasta | 0.10450 | 2.18E-274 | 59/1000 |
| EludwigiiEcWSU1.fasta | SGAir0282.fasta | 0.10526 | 1.71E-269 | 58/1000 |
| EludwigiiEN-119.fasta | SGAir0282.fasta | 0.10526 | 3.56E-269 | 58/1000 |
| EludwigiiI140.fasta | SGAir0282.fasta | 0.10300 | 9.24E-285 | 61/1000 |
| EludwigiiI42.fasta | SGAir0282.fasta | 0.10300 | 9.24E-285 | 61/1000 |
| EludwigiiJP6.fasta | SGAir0282.fasta | 0.10300 | 7.23E-285 | 61/1000 |
| EludwigiiJP9.fasta | SGAir0282.fasta | 0.10300 | 7.23E-285 | 61/1000 |
| EludwigiiP101.fasta | SGAir0282.fasta | 0.10605 | 6.26E-263 | 57/1000 |
| EludwigiiUW5.fasta | SGAir0282.fasta | 0.10450 | 4.10E-274 | 59/1000 |
| EnterobacterODB01.fasta | SGAir0282.fasta | 0.09438 | 0 | 74/1000 |
| EroggenkampiiECY546.fasta | SGAir0282.fasta | 0.07746 | 0 | 109/1000 |
| EroggenkampiiFDAARGOS_523.fasta | SGAir0282.fasta | 0.07630 | 0 | 112/1000 |
| LadecarboxylataUSDA-ARS-USMARC-60222.fasta | SGAir0282.fasta | 0.15662 | 8.63E-81 | 19/1000 |
| EcloacaeA1137.fasta | EsichuanensisWCHECL1597.fasta | 0.02048 | 0 | 482/1000 |

**Additional File: Table S2** Genomic distances estimated by MASH software. Pairwise distance of Reference sequence (Ref seq) against Query sequence (Query seq) was estimated. Dataset defined in the method section was used. Default setting was used for this analysis.
